# Linguistic barriers and healthcare in China: Chaoshan vs. Mandarin

**DOI:** 10.1186/s12913-022-07744-6

**Published:** 2022-03-22

**Authors:** Dangui Zhang, Zichun Jiang, Yu Xie, Weiming Wu, Yixuan Zhao, Anqi Huang, Tumei Li, William Ba-Thein

**Affiliations:** 1 Research Center of Translational Medicine, Second Affiliated Hospital of Shantou University Medical College, Shantou, P.R. China; 2 Undergraduate Research Training Program (UGRTP), Shantou University Medical College, Shantou, P.R. China; 3 Clinical Research Unit, Shantou University Medical College, Xinling Road 22, Shantou, 515041 P.R. China; 4 Department of Microbiology and Immunology, Shantou University Medical College, Xinling Road 22, Shantou, 515041 P.R. China

**Keywords:** Language barrier, Mandarin, Chaoshan, Healthcare communication, Healthcare provider, Healthcare consumer

## Abstract

**Background:**

China has 129 dialects with Mandarin as the standard and Chaoshan as the major dialect of the Chaoshan region in Guangdong. This study aimed to describe the dialect competence and usage, communication difficulty, impact of linguistic barriers, and subjective experience in healthcare.

**Methods:**

Healthcare providers (*n* = 234) and healthcare consumers (*n* = 483) at two tertiary teaching hospitals in Shantou, Chaoshan region participated in an anonymous survey.

**Results:**

Chaoshan and Mandarin were spoken respectively by ca. 80% and 6.1% of the participants. Monolinguals accounted for 28.5%, including 16.8% of Chaoshan-speaking healthcare providers and 18% of Mandarin-speaking healthcare consumers. The monolinguals preferentially used their competent dialect (*Ps* < 0.001) and had significant communication difficulties (*Ps* < 0.0001), with the mean (SD) score of 3.06 (0.96) out of 4 with Mandarin for healthcare providers and 2.18 (1.78) and 1.64 (1.40) with Mandarin and Chaoshan, respectively, for healthcare consumers. The monolingual healthcare providers perceived significant negative impacts of linguistic barriers on the entire healthcare delivery process (*Ps* < 0.0001). Regression analyses showed the length of stay in the Chaoshan region as a protective factor of linguistic barrier with a limited protective effect.

**Conclusions:**

This is the first report of significant linguistic barriers in healthcare imposed by Mandarin and Chaoshan dialects in Chaoshan, China. With perceived adverse impacts on the entire healthcare delivery and risks to the healthcare quality and burden, interventions such as professional interpreter service, service-learning interpreter program, or mobile interpreting apps that are medically accurate and culturally sensitive are suggested for dialectally diverse China.

**Supplementary Information:**

The online version contains supplementary material available at 10.1186/s12913-022-07744-6.

## Background

Culture and linguistic competence affect how people communicate, understand, and respond to information acquired. A linguistic barrier can arise when people speak different languages, different dialects in the same language, and the same language or dialect with different accents. In the healthcare sector, culture and linguistic incompetence can compromise effective healthcare delivery, healthcare consumption, and health outcomes [1]. Patients with linguistic barriers are subject to unnecessary health services, undesirable outcomes, and excess healthcare costs [1, 2].

There are reports about the adverse health impacts from linguistic barriers due to multilingualism (the presence of more than one language) in various communities and regions, including China [3–10], however, such impacts due to multi-dialectalism (the presence of multiple dialects that are mutually unintelligible) are unknown. Because of its multi-dialectalism and multi-accentuality (the presence of variable accents for the same language or dialect), China is at risk of linguistic barriers in its healthcare. But our literature search in the PubMed and Web of Science using keywords “dialect barrier, healthcare, China” as of May 2021 did not turn out any publication.

In China, there are 10 major dialects [11], viz., Mandarin, Wu, Xiang, Yue, Hakka, Gan, Min, Jin, Hui, and Pinghua/Tuhua [12], representing 129 minor dialect variants that are mutually unintelligible [13]. Mandarin is the most widely spoken Chinese dialect by 80.72% of the population in mainland China [14]. The standard Mandarin accent that derives from Beijing Mandarin is uncommon outside Beijing; therefore, Mandarin is spoken in a variety of accents throughout the country.

In Chaoshan, a culturally and linguistically distinct region in the east of Guangdong province in China, the regional dialect is Chaoshan which belongs to the Min major dialect. Although Chaoshan and Mandarin share a common writing system they are different in grammar, vocabulary, and pronunciation.

Chaoshan dialect is considered one of the most difficult Chinese dialects to master because it has eight tones compared to the four tones found in Mandarin. Chaoshan dialect was reportedly spoken by more than 70% of the 14.4 million population in the Chaoshan region in 2018 [15].

Shantou is one of the 3 prefectural cities in the Chaoshan region with a population of 5.64 million as of 2018 and served by 52 hospitals, 19,819 physicians, and 11,011 nurses [16]. Our onsite observations and preliminary investigation including focus group interviews with physicians, nurses, and patients exposed the widespread use of Chaoshan dialect, the presence of communication barriers in clinical encounters, and tolerance by the public as well as healthcare personnel as a cultural norm in Shantou city (unpublished data). These communication barriers in healthcare institutions across the region without formal language support may have an undesirable impact on healthcare delivery and patient-healthcare provider relationship.

This study was aimed at describing the linguistic barrier and its impact on healthcare delivery and consumption in China by investigating the situation in Shantou hospitals in the Chaoshan region.

## Methods

### Study design/site

This cross-sectional study involved a self-developed, anonymized, structured questionnaire-based, self-reported and investigator-assisted survey with a convenience sample of healthcare providers (physicians, nurses, and interns) and healthcare consumers (inpatients, outpatients, and their relatives) at two tertiary teaching hospitals affiliated to Shantou University Medical College in Shantou, Guangdong. These hospitals manned by 1437 physicians and 2281 nurses accommodate a total of 3076 beds [17, 18]. Approximately 0.3% of the city population of 5.64 million in 2018 was accounted for by migrants including healthcare workers [19].

### Survey instrument

One structured questionnaire each was designed for healthcare consumers and healthcare providers to collect demographics, linguistic competence, frequency of usage, degree of communication difficulty, the impact of linguistic barriers, and personal experiences. The survey instruments were validated for the content by two experts before pilot testing with volunteer clinicians and patients for usability and technical functionality.

### Linguistic assessment

**Fig. 1:**
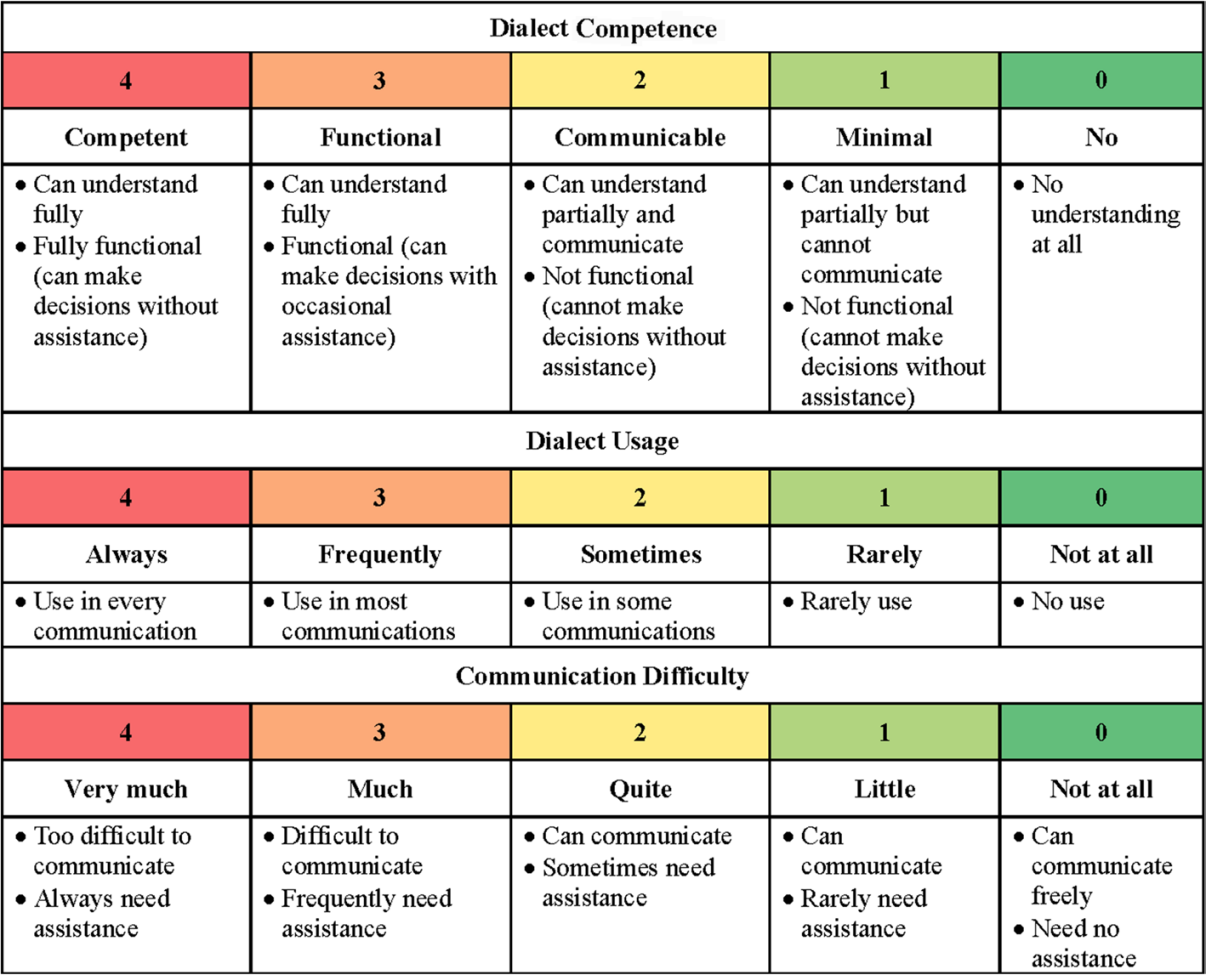
Scales for self-perceived linguistic assessment

Linguistic assessment scales with a range of 0–4 for dialect competence, dialect usage, and communication difficulty (Fig. 1) were self-developed based on focus group interviews with clinicians and patients.

### Survey administration

With permission from the departmental heads of the participating hospitals, trained study staff (a pair of Mandarin and bilingual speakers) approached and requested patients and accompanies for participation in the questionnaire survey. When needed, the study staff assisted them to complete the questionnaire.

### Data analysis

Data from collected surveys were transferred into a database and cross-checked by two study staff and analyzed using SPSS (version 20). Participants with a language competence score ≥ 3 in both Mandarin and Chaoshan were classified as bilinguals. Five-point Likert scales (0–4) were treated as continuous data and presented as mean scores. Other continuous variables including age, work experience, and living time in the Chaoshan region were shown as the median and interquartile range (IQR), and categorical variables including gender, birthplace, mother tongue, competent dialect, education, and job position as number and percentage. T-test or one-way ANOVA was used for the analysis of continuous variables, Pearson’s correlation for correlation between continuous variables, and multiple linear regression for perceived communication difficulty in healthcare delivery, with consideration of a two-tailed *P*-value of < 0.05 as statistical significance.

## Results

### Participant characteristics (Table 1)

There were 717 study participants, including 234 healthcare providers (doctors, nurses, and interns) and 483 healthcare consumers (146 patients and 337 of their relatives). The median age of the participants was 31 for the healthcare providers and 36 for the healthcare consumers. Male to female ratio was 0.34 for the healthcare providers and 1.06 for the healthcare consumers. The median working year of healthcare providers was 6 years in the current hospital and 7.3 years in the healthcare sector, with 93.6% (219/234) being experienced practicing clinicians. The median living time in the Chaoshan region was 27.8 years for healthcare providers and 30 years for healthcare consumers. Most healthcare consumers (71.2%, 344/483) had below high school education.

**Table 1:** Demographics of study participants (*N* = 717)

Characteristics	Healthcare Providers^a^*n* = 234	Healthcare Consumers^b^*n* = 483
***Continuous variables: median (IQR)***		
Age (year)	31 (26–35)	36 (29–50)
Work experience (month)		
Current Department	41 (14–90)	NA
Current Hospital	72 (27–120)	NA
Healthcare	88 (36–136)	NA
Living time in Chaoshan region (month)	334 (276–396)	360 (264–549)
***Categorical variables: n (%)***		
Sex		
Male	60 (25.6)	249 (51.6)
Female	174 (74.4)	234 (48.4)
Birthplace		
Chaoshan region	196 (83.8)	387 (80.1)
Non-Chaoshan region	38 (16.2)	96 (19.9)
Mother tongue (Native or first language)		
Mandarin	21 (9.0)	23 (4.8)
Chaoshan	192 (82.0)	386 (79.9)
Others^c^	21 (9.0)	74 (15.3)
Competent dialect		
Mandarin	36 (15.4)	87 (18.0)
Chaoshan	0 (0)	81 (16.8)
Bilingual^d^	198 (84.6)	315 (65.2)
Education		
Primary school or lower	NA	101 (20.9)
Middle school	NA	243 (50.3)
High school	NA	72 (14.9)
College or higher	NA	67 (13.9)
Job Position		
Chief Physician/Nurse	1 (0.4)	NA
Assoc. Chief Physician/Nurse	9 (3.8)	NA
Assist. Physician/Nurse-in-charge	70 (29.9)	NA
Resident/Nurse	139 (59.4)	NA
Intern	15 (6.4)	NA

^a^doctors, nurses, and interns

^b^patients and their relatives

^c^including 17 additional dialects

^d^having a language competence score ≥ 3 in both Mandarin and Chaoshan

### Dialect competence and usage in clinical encounters (Table 1 and Fig. 2)

Overall, 19 dialects were identified native to the study participants. The majority (ca. 80%) of both healthcare consumers and providers were born in the Chaoshan region with the Chaoshan dialect as their first language or mother tongue. Mandarin was native to 6.1% (44/717), and 17 additional dialects were native to the remaining participants (13.2%, 95/717).

**Fig. 2:**
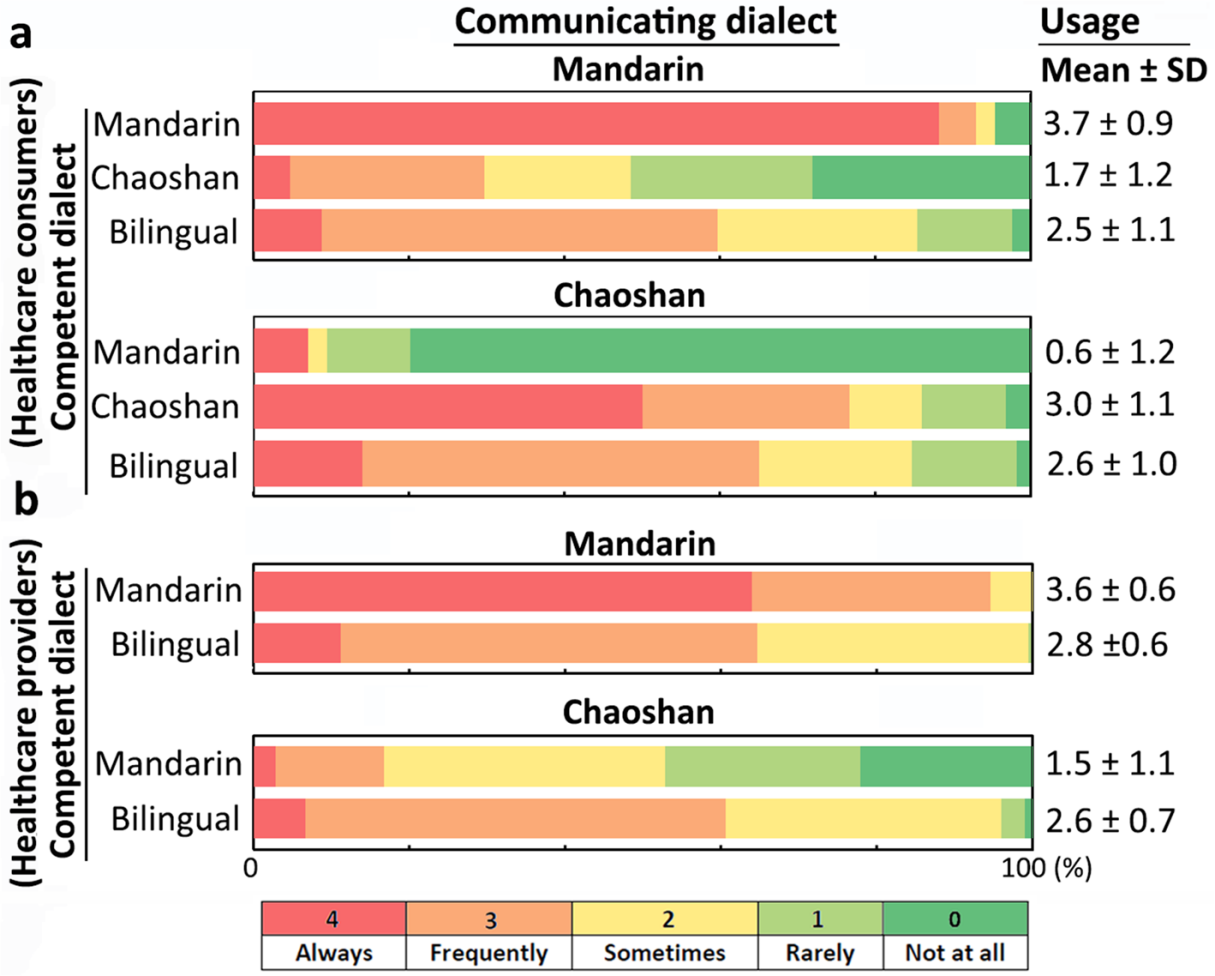
Dialect usage frequency of (**a**) healthcare consumers and (**b**) healthcare providers during clinical encounters

Based on the competence of Mandarin (the standard dialect) and Chaoshan (the major regional dialect), we categorized the healthcare consumers into Mandarin, Chaoshan, and bilingual speakers, and the healthcare providers into Mandarin and bilingual speakers because all Chaoshan-speaking healthcare providers spoke Mandarin.

Those who had a dialect competence score of 4 only in Mandarin or Chaoshan were reported herein as competent for the respective dialect and who scored ≥3 in both Mandarin and Chaoshan as bilinguals.

Among the healthcare consumers, 16.8% (81/483) spoke Chaoshan only, whereas 15.4% (36/234) of healthcare providers and 18% (87/483) of healthcare consumers spoke Mandarin only, resulting in 28.4% (204/717) of monolinguals among the study participants (Table 1).

Figure 2 shows self-reported competence and usage of dialects by healthcare providers and consumers during clinical encounters. The monolinguals preferentially used their competent dialect (*Ps* < 0.001), whereas the bilinguals used Chaoshan and Mandarin equally (mean ± SD, 2.6 ± 0.7 vs 2.8 ± 0.6, *P* = 0.054 for the healthcare providers; 2.6 ± 1.0 vs 2.5 ± 1.1, *P* = 0.454 for the healthcare consumers).

**Fig. 3:**
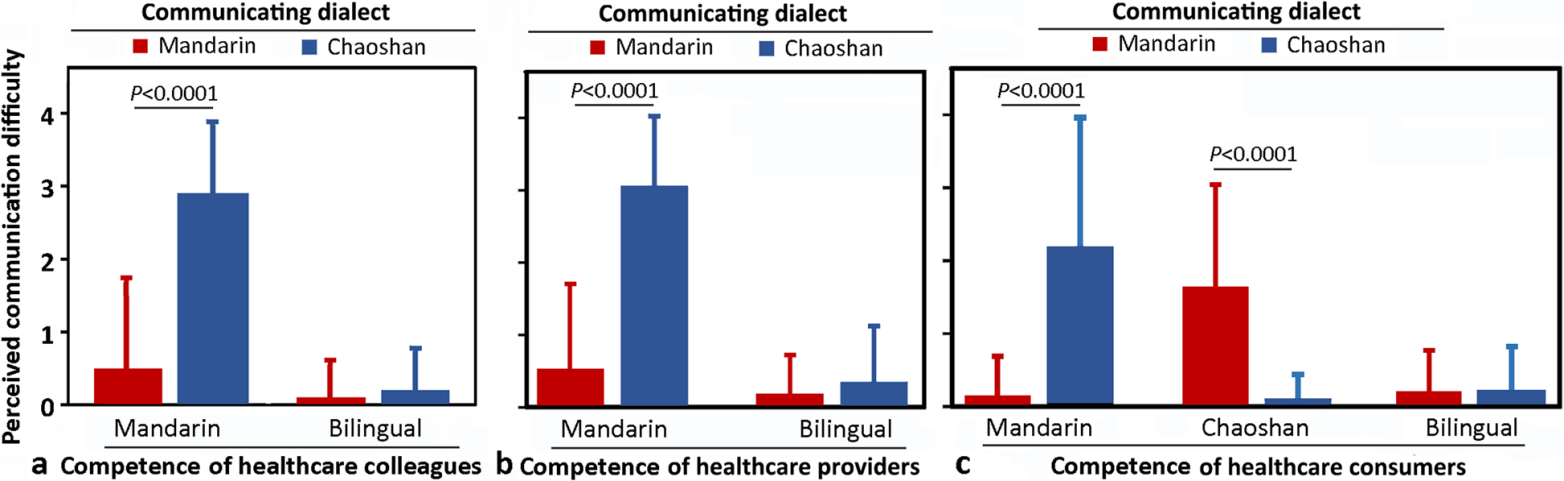
Perceived communication difficulties due to dialect barriers in (**a**) healthcare communication, **b** healthcare delivery, and **c** healthcare consumption. *P* values were analyzed by t-test

Perceived communication difficulties in healthcare delivery and consumption (Fig. 3 and Supplementary Tables 1 and 2).

Significant difficulties existed among the healthcare providers (i.e., between physicians, between nurses, and between physicians and nurses) during clinical communication and teamwork, with a linguistic difficulty score of 2.9 ± 1.0 (mean ± SD) (*P* < 0.0001 by t-test in Fig. 3a; *P* < 0.001 by t-test in Supplementary Table 1).

During healthcare delivery, both the monolingual healthcare providers and consumers had significant difficulties when communicating in their non-competent dialect during clinical encounters, with a linguistic difficulty score of 3.1 ± 1.0 (mean ± SD) with Mandarin for the healthcare providers and 2.2 ± 1.8 and 1.6 ± 1.4 with Mandarin and Chaoshan, respectively, for the healthcare consumers (*Ps* < 0.0001 by t-test in Fig. 3b and c; *Ps* < 0.001 by t-test or ANOVA in Supplementary Table 2). The bilinguals did not have any significant difficulty.

In response to an open question about their subjective experiences, 7.5% (54/717) of the participants, including 14 patients and 40 clinicians, mentioned lack of competence and variable accents of both Mandarin and Chaoshan speakers as the major barriers. Common communication difficulties they had were due to monolingual elderly patients, patients speaking Hakka dialect (the major dialect of the nearby regions such as Meizhou city), and older physicians (data not shown).

### Impact of dialect barriers in healthcare communication, delivery, and consumption (Fig. 4)

**Fig. 4:**
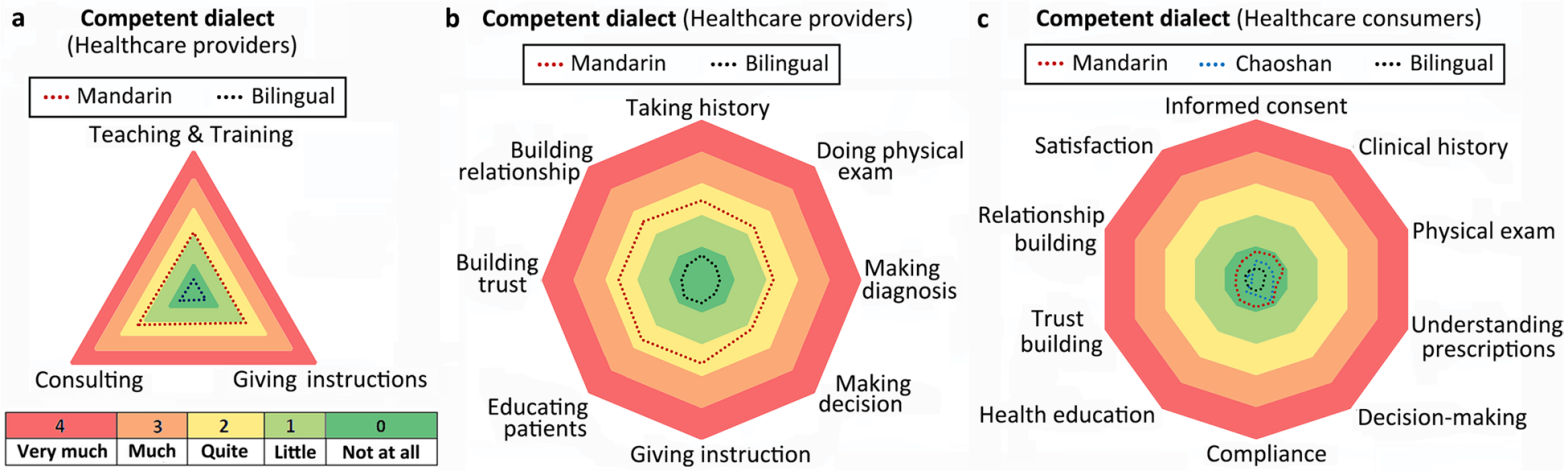
Perceived negative impact on (**a**) communication with colleagues, **b** healthcare delivery, and **c** healthcare consumption due to dialect barriers

The Mandarin monolingual healthcare providers perceived significant negative impacts on interprofessional interaction and communication such as teaching, training, instruction, and giving consultations to colleagues (*Ps* < 0.0001, Fig. 4a). Perceived negative impact from the dialect barrier was significantly greater in the Mandarin monolinguals than the bilingual healthcare providers for the entire healthcare delivery process, i.e., taking clinical histories, doing physical exams, making diagnoses and clinical decisions, educating patients, and building trust and relationships with patients (*Ps* < 0.0001, Fig. 4b). Regardless of dialect competence and communication difficulties, the healthcare consumers, however, did not perceive any significant negative impact while receiving healthcare (mean ± SD, 0.4 ± 0.1, Fig. 4c).

### The dynamics of dialect barrier over time with healthcare providers (Fig. 5 and Table 2)

**Fig. 5:**
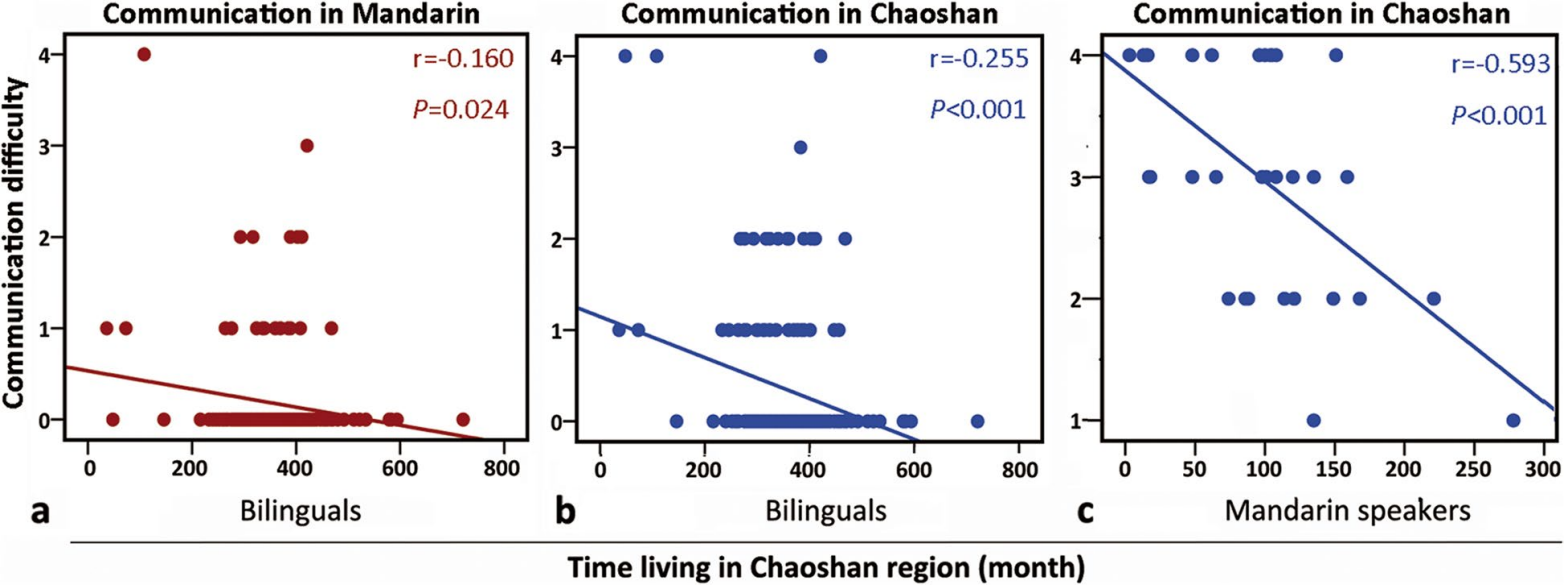
The dynamics of communication difficulty over time with (**a**) Mandarin and (**b and c**) Chaoshan among healthcare providers, analyzed with Pearson’s correlation

Since healthcare providers are primarily responsible for effective health communication, we explored potential aspects for intervention by analyzing their demographics vs. linguistic difficulties. Living time in the Chaoshan region had negative relationships with the dialect barrier, with a weak correlation with Mandarin (*r* = − 0.160, *P* = 0.024, Fig. 5a) and Chaoshan (*r* = − 0.255, *P* < 0.001, Fig. 5b) for the bilinguals and a moderate correlation with Chaoshan (*r* = − 0.593, *P* < 0.001, Fig. 5c) for the Mandarin monolinguals by Pearson’s correlation analysis.

**Table 2:** Multiple linear regression analysis of perceived communication difficulty in healthcare delivery

	Communicating in Mandarin			Communicating in Chaoshan		
	Beta	95%CI	*P*	Beta	95%CI	*P*
**For Mandarin-speaking healthcare providers**						
Time living in Chaoshan region (mo)^a^	- 0.002	- 0.012 ~ 0.009	0.745	- 0.013	- 0.20 ~ - 0.006	< 0.0001
Being male	0.029	- 0.910 ~ 0.968	0.950	0.337	- 0.267 ~ 0.940	0.264
Older age (year)^a^	0.072	- 0.060 ~ 0.204	0.276	0.070	- 0.014 ~ 0.155	0.101
Higher rank of job position^b^	0.792	- 0.082 ~ 1.665	0.074	0.355	- 0.206 ~ 0.917	0.207
**For Bilingual healthcare providers**						
Time living in Chaoshan region (mo)	- 0.002	- 0.003 ~ -0.001	0.001	- 0.003	- 0.005 ~ - 0.002	< 0.0001
Being male	0.003	- 0.175 ~ 0.181	0.974	0.010	- 0.238 ~ 0.259	0.936
Older age (year)^a^	0.020	- 0.002 ~ 0.042	0.074	0.029	- 0.001 ~ 0.060	0.060
Higher rank of job position^b^	- 0.030	- 0.183 ~ 0.123	0.703	0.088	- 0.125 ~ 0.302	0.415

^a^continuous variable

^b^ordinal variable (chief physician/nurse, associate physician/nurse, assitant physician/nurse-in-charge, resident/nurse, intern)

The variables of significant correlation were included in multiple linear regression analysis (Table 2). After controlling for age, gender, and job position, there were significant negative directional relationships between living time in the Chaoshan region and the communication difficulty with Chaoshan (beta - 0.013 for the Mandarin-speakers and - 0.003 for the bilinguals; *Ps* < 0.0001) or Mandarin (beta - 0.002 for the bilinguals, *P* < 0.001).

## Discussion

This study is the first of its kind to present evidence for widespread use of the local dialect Chaoshan in healthcare, communication barriers due to both the Mandarin and Chaoshan, and significant adverse impacts on healthcare communication, delivery, and consumption in the linguistically diverse Chaoshan region. Although the dialect barriers to healthcare in this study represent only the Chaoshan region, similar situations could be expected in other regions of China where Mandarin is not the competent dialect.

### Dialectal preference during clinical encounters

Being the official common language of China’s nine-year compulsory education that came into effect in 1986 [20], Mandarin is spoken ubiquitously, albeit with geographical accents, by well-educated and young Chinese across China. However, 400 million people especially older Chinese and those in rural areas cannot speak Mandarin [21]. In the Chaoshan region, the local dialects are considered socially obligatory and thus used extensively even in health services. A considerable proportion of older Chaoshanese (16.8%, 81/483 of healthcare consumers in this study with a mean age of 67 years) were monolingual Chaoshan speakers.

For the native speakers of Mandarin (6.1%, 44/717) and the migrants from various provinces with their own dialects that are mutually unintelligible (13.2%, 95/717), Mandarin became their preferred dialect for communication in the Chaoshan region.

### Impact on healthcare delivery and consumption due to communication difficulties

Healthcare is teamwork and thus communication with clarity and continuity among the team members is indispensable for delivering quality healthcare and patient safety. The dialect barriers, however, appeared to have impaired effective health communication not only between the healthcare providers and consumers but among the healthcare colleagues as well in this study.

Health outcomes can be adversely affected by poor compliance from unpleasant clinical encounters if patients feel their voices are not heard or they are not respected [22]. The communication barrier impacted clinical encounters at emergency, ambulatory, and inpatient care in this study. The monolingual speakers of Mandarin or Chaoshan had significant communication difficulties in delivering or consuming healthcare (Fig. 3) due not only to discordant dialects but also to the variable accents, which could inevitably lead to ad hoc interpretation by whoever accompanying them.

The perception of adverse impact on the entire healthcare delivery process is alarming because these monolinguals represented 15.4% of providers, thus indicating the presence of compromised quality healthcare, including the patient-clinician relationship, trust, compliance, and the overall health outcome. Such indication is supported by the subjective experiences of clinicians as:

*“A Chaoshan-speaking patient and her companion visited the EMD at a late-night hour. I got panic as none of us could understand them because they spoke Chaoshan only; I had to call one off-duty nurse for help. That 15-20-min waiting time was terrible and so risky.” (Physician in-charge, Emergency Dept.)*

*“I was unable to communicate with a patient under critical condition because my senior who could speak Chaoshan was not with me.” (Young physician)*

“*I am confused every day during ward rounds. My doctor-patient communication skill is zero.*” *(Young physician)*

“*I was refused directly by the patients for any medical diagnosis or treatment because I cannot speak the local dialect.*” *(Migrant physician)*

“*I cannot explain the professional terms in Chaoshan*.” *(Bilingual physician)*

“*I have a lot of pressure from the widespread use of Chaoshan dialect at work.*” *(Newly appointed clinician)*

“*Patients didn’t understand and cooperate with me for physical exams.” (Clinician)*

Since clinical communication is a two-way interaction between the patients and clinicians, it was unexpected and interesting to find that they differed significantly in perceiving the dialect impact. While the clinicians recognized the dialect barriers as a jeopardy to the entire healthcare delivery process, the patients felt none on the receiving end.

There are certain conceivable reasons for the reported no negative impact by the healthcare consumers. Monolingual patients are usually accompanied by family members who would not only break the language barrier but also ease any impact negative on the patients. Also, limited education, which is associated with low health literacy, in more than 70% of our healthcare consumers might have blinded them from sensing or perceiving the impact that the communication barrier had on health services. Most importantly, the ordinary Chinese people, and even clinicians, are uninformed of common healthcare-related concepts such as patient values or quality healthcare and thus oblivious about their rights (unpublished data from our communication with clinical trainees and patients).

Amid escalating doctor-patient tensions in China, poor communication is one of the blamed factors in building relationships and trust [23], but our study revealed that communication difficulty does not always translate into a poor relationship or trust-building in the context of the patients` perspective. Clinician’s professionalism such as attitudes and behaviors other than the dialect barrier could be more important for the patients as some of them in this study said:

*“The doctor spoke something unclearly and I didn't understand; when I asked him to repeat, his attitude became bad.”*

*“The staff only spoke the local dialect and was impatient to explain when asking for more explanation.” “Communication is not convenient, and I felt discriminated by the local culture”.*

On the other hand, miscommunication as a psychological stressor was reported by one clinician:

*“I may have made the patient’s relatives misunderstand that I am impatient to give them instructions.”*

Therefore, as discussed previously [22], miscommunication could be a potential flashpoint of patient-clinician conflict from misunderstanding. The study participants` subjective stories could be just the tip of the linguistic problems in ensuring quality healthcare, such as excess healthcare services, health burden, and healthcare cost, and compromised patient safety as reported before [1, 2].

### Exposure time to the second language (L2) and communication difficulty for healthcare providers

Some clinicians were simultaneous bilingual speakers (who grew up speaking Chaoshan and Mandarin) and some were sequential bilinguals (who acquired either Mandarin or Chaoshan as L2 later in school or after migration to the Chaoshan area). We found that the clinicians who had been living in the Chaoshan region for more than 20–30 years still could not overcome the linguistic barrier.

Our regression analyses showed that the length of stay in the Chaoshan region was a protective factor of the linguistic barrier, but the protective effect is very limited as it could take considerably lengthy years to break the linguistic barrier. Although many factors, such as age, motivation, or opportunity, can influence L2 acquisition for busy clinicians, acquiring competence of another dialect for clinical communication is not practicable.

### Interventions for overcoming dialect barriers to healthcare in China

Most Chinese hospitals have a kind of hospital visitor assistant system with patient escorts/ushers. But these escorts assist visitors with the directions only; therefore, dialect barrier problems remain unattended. For instance, one patient in this study said: “*I am scared of going to hospitals because I feel nervous and under pressure to communicate with clinicians; I couldn’t describe clearly my illness and also couldn’t understand the explanation of doctors*.”

All the patients in this study were accompanied by their families or relatives, some as young as 17 years old. It is not uncommon for language-disadvantaged patients to have family members or friends and even minors or strangers as interpreters in clinical situations, but there is a risk of information distortion or inveracity and compromised patient-centeredness, clinician-patient relationship, and confidentiality. Untrained ad hoc interpreters are susceptible to inserting or omitting information, infusing personal opinions and assumptions, leaving the patients out of discussions, or even providing informed consent or making decisions on behalf of the patients for sociocultural or emotional reasons during clinical interactions [2, 24–26].

There are well-recognized breakers of language barriers to healthcare [1, 2], such as professional interpreting service [27–29], language training of healthcare providers [30], or medical translation apps [31, 32], which are nonetheless relevant mainly to LEP (Limited English Proficiency) in recent and remote immigrants in English-speaking resource-rich countries like the United States, Australia, and United Kingdom [29, 31–33]. The recommended interventions are, therefore, far to be practical in dialectically diverse countries like India [9] or China. For example, on-site professional interpreter service is recommended most for its overall effectiveness [24, 28], but its cost [27, 33, 34] and in-house after-hours service could be significant concerns for Chinese hospitals because even public hospitals in China are only partially funded by the government [35].

Training bilingual hospital employees such as nurses and technicians for ad hoc medical interpretation [2, 26] is another logical approach; nevertheless, this could also conflict with the understaffing problem in most Chinese hospitals.

Recruiting and training retired bilingual nurses, who generally have rich clinical experiences, for on-call interpreting service could be a viable option in China. Alternatively, we propose teaching hospitals in China to implement a service-learning interpreter program for bilingual senior medical and nursing students who do not need to acculturate to local socio-cultural sensitivity. Such a program could be introduced as an experiential learning elective to reap the students` service while preparing them to become competent clinical communicators.

Recently, Internet and mobile apps have become popular translation aid among healthcare personnel and patients [32, 36]. Although they could alleviate language barriers during low-risk clinical communications, they are not a suitable substitute for trained interpreters due to major concerns as to translation accuracy and cultural appropriateness [32]. Given the integration of translation technology in healthcare becoming imminent, it is worth exploring such technological applications in the Chinese healthcare context.

### Study limitations

Having to maintain anonymity, we neither collected the participants` identifiers nor followed them and thus were unable to investigate relationships between healthcare quality and linguistic barrier or impact perception. Particularly, disagreeing perception of linguistic impact by the healthcare providers and consumers should be further explored in the context of health burden, health outcome, and patient safety. Besides, older patients and clinicians, who are at risk of language difficulties, should be included in future studies to understand real-life situations.

## Conclusions

This study in Chaoshan has demonstrated for the first time that incompetence and variation in the accents of the standard as well as local dialects impose significant linguistic barriers to healthcare in China. In consideration of perceived adverse impacts on the entire healthcare delivery and predictable risks to the healthcare quality and burden, interventions such as professional interpreter service or a service-learning interpreter program for clinical students in teaching hospitals are strongly suggested. It is also worth developing mobile interpreting apps that are medically accurate and culturally sensitive for dialectally diverse China.

## Supplementary Information

**Additional file 1: Suppl Table 1.** Perceived communication difficulty in healthcare communication. **Suppl Table 2.** Perceived communication difficulty in healthcare delivery and consumption.

## Data Availability

The datasets generated and analyzed during the current study are available from the corresponding author on reasonable request.
