# Supplementary material for: Linguistic barriers and healthcare in China: Chaoshan vs. Mandarin
Source: BMC Health Serv Res. 2022 Mar 22;22:376. doi: 10.1186/s12913-022-07744-6 (PMC8941784; doi:10.1186/s12913-022-07744-6)
Supplement: Supplementary file 1 — Additional file 1: Suppl Table 1. Perceived communication difficulty in healthcare communication. Suppl Table 2. Perceived communication difficulty in healthcare delivery and consumption. [file 12913_2022_7744_MOESM1_ESM.docx]

**Suppl Table 1. Perceived communication difficulty in healthcare communication**

| **Competent dialect** | Total (N = 234) | **Communicating dialect** | | |  |
| --- | --- | --- | --- | --- | --- |
|  |  | **Mandarin** |  | **Chaoshan** |  |
| *Healthcare colleagues* |  |  | | | |
| Mandarin | 36 (15.4) | 0.5 ± 1.2 |  | 2.9 ± 1.0 | *P* <.001^1^ |
| Bilingual | 198 (84.6) | 0.1 ± 0.5 |  | 0.2 ± 0.6 |  |

Data shown as language difficulty score (mean ± SD); see methods for details. ^1^ analyzed by t-test.

**Suppl Table 2. Perceived communication difficulty in healthcare delivery and consumption**

| **Competent dialect** | Total (N = 717) | **Communicating dialect** | | |  |
| --- | --- | --- | --- | --- | --- |
|  |  | **Mandarin** |  | **Chaoshan** |  |
| *Healthcare providers* | n = 234 (%) |  | | | |
| Mandarin | 36 (15.4) | 0.5 ± 1.2 |  | 3.1 ± 1.0 | *P* <.001^1^ |
| Bilingual | 198 (84.6) | 0.2 ± 0.5 |  | 0.4 ± 0.8 |  |
| *Healthcare consumers* | n = 483 (%) |  | | | |
| Mandarin | 87 (18.0) | 0.2 ± 0.5 | *P* <.001^2^ | 2.2 ± 1.8 | *P* <.001^2^ |
| Chaoshan | 81 (16.8) | 1.6 ± 1.4 |  | 0.1 ± 0.3 |  |
| Bilingual | 315 (65.2) | 0.2 ± 0.6 |  | 0.2 ± 0.6 |  |

Data shown as language difficulty score (mean ± SD); see methods for details. ^1^ analyzed by t-test; ^2^ analyzed by one-way ANOVA.
